# Disjonction symphysaire après un accouchement par voie basse dystocique: à propos d'un cas

**DOI:** 10.11604/pamj.2014.17.33.3441

**Published:** 2014-01-18

**Authors:** Meriem Laadioui, Wafae Slimani, Sofia Jayi, Fatimazahra Fdili Alaoui, Hakima Bouguern, Hikmat Chaara, Moulay Aabdelilah Melhouf

**Affiliations:** 1 Service de Gynécologie-Obstétrique 2, Chu Hassan II De Fès, Université Sidi Mohammed Ben Abdellah, Maroc

**Keywords:** Disjonction symphysaire, accouchement dystocique, rupture uterine, symphysis disjunction, obstructed labor, uterine rupture

## Abstract

La disjonction symphysaire est une affection rare, qui se définie par un élargissement au niveau de l'articulation inter-symphysaire estimé supérieur à 10 mm. Cette affection nécessite une prise en charge spécialisée en cas de douleurs sévères et invalidantes. Nous rapportant le cas d'une patiente présentant des douleurs pelviennes intense avec impotence du MI gauche à J2 d'un accouchement dystocique, l'examen clinique a objectivé une douleur exquise à la palpation de la symphyse pubienne. Le diagnostic a été confirmé par une radiologie du bassin de face objectivant un élargissement de la symphyse pubienne de 15 mm, la prise en charge thérapeutique a consisté en une mise sous décharge et anti-coagulation préventive avec un traitement antalgique à base de paracétamol et AINS. L’évolution était favorable. A travers notre cas, nous insisterons sur les caractéristiques de cette pathologie notamment pronostic, ce qui permettra au praticien de comprendre l'intérêt du diagnostic et prise en charge précoce de cette entité qu'elle évoquer devant toute douleurs pelviennes survenant au cours de la grossesse ou en post partum.

## Introduction

Le diagnostic d'un syndrome de disjonction symphysaire est évoqué cliniquement devant des douleurs insidieuses survenant chez la femme enceinte ou brutalement en post-partum, peut être confirmé par une radiologie de bassin face montrant un espace inter-symphysaire supérieur à 10 mm. La prise en charge initiale doit être précoce afin d'assurer une autonomie et un confort à la parturiente. Nous rapportons l'observation d'une patiente ayant présenté un syndrome de disjonction symphysaire suite à un accouchement dystocique.

## Patient et observation

**Figure 1:**
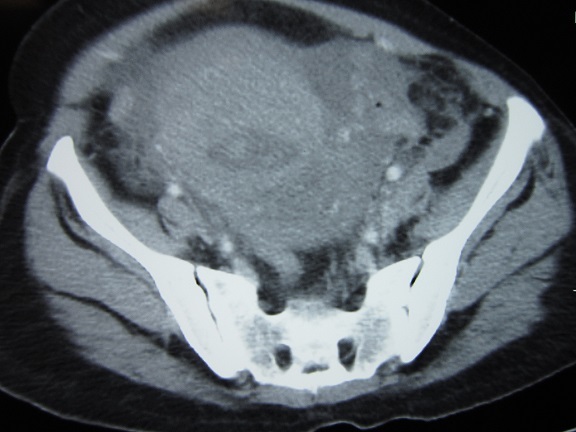
Aspect de rupture utérine sous péritonéale associé à la disjonction symphysaire

**Figure 2:**
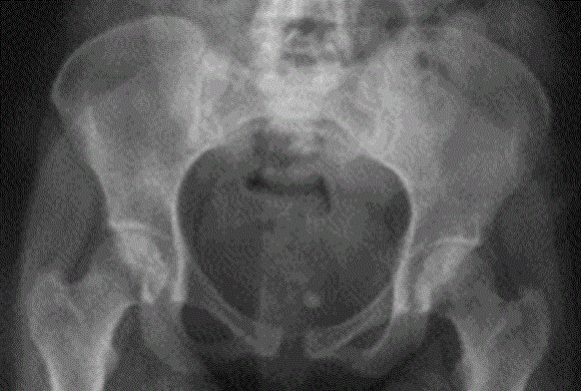
Radiologie du bassin de face objectivant un élargissement de l'espace inter symphysaire

Patiente de 38 ans, cinquième geste cinquième pare, la grossesse actuelle n'a pas été suivie, déroulement apparemment normal, notamment pas de notion de douleurs pelviennes, cette grossesse a été menée à terme avec tentative d'accouchement par voie basse à domicile, admise dans notre formation 5h après pour rétention des épaules, la patiente a été admise au bloc opératoire, après la mise en condition, le dégagement des épaules fait par manoeuvre de Mc Roberts avec issue d'un mort-né de 4900g, l'examen sous valve a objectivé une déchirure de la commissure cervicale gauche qui a été suturée. A J2 du post partum, la patiente a présenté des douleurs abdomino-pelviennes intenses sans irradiation particulière avec impotence du MI gauche. L'examen clinique a objectivé une sensibilité abdomino-pelvienne plus importante à la palpation de la symphyse pubienne. La TDM abdomino-pelvienne a révélé une rupture utérine sous péritonéale avec un hématome qui fuse jusqu'au ligament lombo-ovarien gauche (Figure 1)qui a été respecté. La radiologie du bassin de face a objectivé un élargissement de la symphyse pubienne de 15 mm (Figure 2), la prise en charge thérapeutique été la mise sous décharge et anti-coagulation préventive avec un traitement antalgique à base de paracétamol et AINS. L’évolution été favorable sans récidive douloureuse et elle a été déclarée sortante à J12 du postpartum. L'examen lors du contrôle réalisé après 15 jours n'objective pas de sensibilité au niveau symphysaire.

## Discussion

L'incidence du syndrome de disjonction pubienne en péripartum est évaluée entre 1/300 et 1/30 000 dans la littérature [1, 2], en effet 22% des parturientes peuvent avoir des douleurs au niveau de la symphyse pubienne, ces douleurs sont atroces chez 5-8% des parturientes. 7% des parturientes ont cette symptomatologie en post partum [3, 4]. Les étiologies de cette affection restent mal connues bien que plusieurs auteurs ont rapporté l'association fréquence de la disjonction symphysaire et certains facteurs de risque notamment la macrosomie foetale, les manoeuvres d'extraction, les pathologies articulaires et les traumatismes de l'articulation pubienne [5, 6]. Notre patiente avait de nombreux facteurs de risque sus-cités à savoir la multiparité, la macrosomie et la pratique de la manoeuvre de Mc robert pour le dégagement des épaules. Le diagnostic repose sur la symptomatologie rapportée par la patiente et l'examen clinique. La symptomatologie typique semble comporter une douleur de la symphyse pubienne avec irradiations inguinales associée à une douleur de l'articulation sacro-iliaque [7]. L'examen clinique note une douleur exquise à la palpation de la symphyse pubienne, voir même un oedème de la symphyse et la palpation vraie d'un espace inter-symphysaire [8]. Le tableau clinique dans notre observation été assez typique. Le diagnostic paraclinique repose sur une radiologie du bassin de face montrant un espace inter-symphysaire supérieur à 10 mm [7], cet espace été évalué à 15 mm dans le cas de notre patiente. Le degré de séparation observé n'apparaît cependant pas corrélé à la gravité des symptômes [5], certains auteurs ont proposé l’échographie comme moyen diagnostic en particulier au cours de la grossesse où une radiographie standard n'est pas autorisé, mais ils concluent au caractère non prédictif de cet examen [9]. Le syndrome de disjonction symphysaire survient souvent en fin de grossesse ou en post partum, des cas ont été rapporté dans la littérature survenant plus précocement à 20 et à 31 SA [6, 7] La prise en charge initiale précoce est médicale avec l'association d'une analgésie par voie orale, du repos et de la kinésithérapie. En cours de grossesse et à un terme précoce, l’échec d'une infiltration locale proposée pourrait justifier la mise en place d'une analgésie péridurale selon certains auteurs [7]. En post-partum, l'infiltration locale au niveau de la symphyse pubienne apparaît efficace. Selon les recommandations elle doit s'effectuer au bloc opératoire en conditions strictes d'asepsie et utiliser une solution associant un anesthésique local et un corticoïde [10]. Dans notre cas, il s'agissait d'une symptomatologie apparue en post-partum immédiat avec des signes radiologiques. Le traitement médical conservateur associant la décharge, antalgiques et anti-coagulation préventive a permis une évolution favorable avec une nette amélioration de la douleur. Notre patiente n'a pas bénéficié d'un bandage pelvien qui apparaît plutôt recommandé en cas de diastasis important [6, 11]. selon la littérature un traitement chirurgical peut être proposé en cas de diastasis supérieur à 4 cm [11].

## Conclusion

Le syndrome de disjonction symphysaire doit être évoqué devant toutes douleurs pelviennes de la grossesse et du post-partum, le diagnostic est posé facilement grâce à une radiologie du bassin de face montrant un espace intersymphysaire supérieur à 10 mm. La prise en charge initiale précoce est médicale avec l'association d'une analgésie par voie orale voir une infiltration locale, du repos et de la kinésithérapie. Le bandage pelvien et le traitement chirurgical sont réservés aux cas de diastasis importants et doivent être associés à une anticoagulation préventive en cas d'immobilisation.

## References

[1] Kubitz RL, Goodlin MD (1986). Symptomatic separation of the pubic symphysis. South Med J..

[2] Williams JW, Eastman NJ, Hellman LM, Lichtman Marshall A (1966). Coincidental complications of pregnancy. Williams obstetrics.

[3] Albert H, Godskesen M, Westergaard J (2001). 2001 Prognosis in four syndromesof pregnancy-related pelvic pain. Acta Obstet Gynecol Scand..

[4] Jain S, Eedarapalli P, Jamjute P, Sawdy R (2006). 2006 Symphysis pubis dysfunction:a practical approach to management. Obstetrician and Gynaecologist..

[5] Snow RE, Neubert AG (1997). Peripartum pubic symphysis separation during pregnancy: a case report. Obstet Gynecol Surv..

[6] Culligan P, Hill S, Heit M (2002). Rupture of the symphysis pubis during vaginal delivery followed by two subsequent uneventful pregnancies. ObstetGynecol..

[7] Scicluna JD, Alderson JD, Webster VJ, Whiting P (2004). Epidural analgesiafor acute symphysis pubis dysfunction in the second trimester. Int J Obstet Anesth..

[8] Luger EJ, Arbel R, Dekel S (1995). Traumatic separation of thrsymphysis pubis during pregnancy: a case report. J Trauma..

[9] Scriven MW, Jones DA, McKnight L (1995). The importance of pubic pain following childbirth: a clinical and ultrasonographic study of diastasisof the pubic symphysis. J R Soc Med..

[10] Waldam SD, Waldam SD, Steven D (2003). Ostéitepubienne. Atlas des syndromes douloureux fréquents - Paris, Maloine.

[11] Kharrazi FD, Rodgers WB, Kennedy JG, Lhowe DW (1997). Parturition-induced pelvic. dislocation: a report of four cases. J Orthop Trauma..

